# Characteristics of student preparedness for clinical learning: clinical educator perspectives using the Delphi approach

**DOI:** 10.1186/1472-6920-12-112

**Published:** 2012-11-13

**Authors:** Lucinda S Chipchase, Peter J Buttrum, Ruth Dunwoodie, Anne E Hill, Allison Mandrusiak, Monica Moran

**Affiliations:** 1 The University of Queensland, School of Health and Rehabilitation Sciences, Queensland, Australia; 2 QE II Jubilee Hospital, Department of Physiotherapy, Queensland, Australia; 3 The University of Queensland, School of Health and Rehabilitation Sciences, Brisbane, 4072, Australia

**Keywords:** Delphi study, Clinical learning, Preparedness

## Abstract

**Background:**

During clinical placements, clinical educators facilitate student learning. Previous research has defined the skills, attitudes and practices that pertain to an ideal clinical educator. However, less attention has been paid to the role of student readiness in terms of foundational knowledge and attitudes at the commencement of practice education. Therefore, the aim of this study was to ascertain clinical educators’ views on the characteristics that they perceive demonstrate that a student is well prepared for clinical learning.

**Methods:**

A two round on-line Delphi study was conducted. The first questionnaire was emailed to a total of 636 expert clinical educators from the disciplines of occupational therapy, physiotherapy and speech pathology. Expert clinical educators were asked to describe the key characteristics that indicate a student is prepared for a clinical placement and ready to learn. Open-ended responses received from the first round were subject to a thematic analysis and resulted in six themes with 62 characteristics. In the second round, participants were asked to rate each characteristic on a 7 point Likert Scale.

**Results:**

A total of 258 (40.56%) responded to the first round of the Delphi survey while 161 clinical educators completed the second (62.40% retention rate). Consensus was reached on 57 characteristics (six themes) using a cut off of greater than 70% positive respondents and an interquartile deviation IQD of equal or less than 1.

**Conclusions:**

This study identified 57 characteristics (six themes) perceived by clinical educators as indicators of a student who is prepared and ready for clinical learning. A list of characteristics relating to behaviours has been compiled and could be provided to students to aid their preparation for clinical learning and to universities to incorporate within curricula. In addition, the list provides a platform for discussions by professional bodies about the role of placement education.

## Background

Entry-level education of students within the allied health professions aims to equip graduates with the required knowledge, skills and professional behaviours to work safely and competently as contemporary health care professionals. At the core of entry-level education is the clinical education program that involves a symbiotic collaboration between clinical providers and universities. Broadly, clinical education involves learning clinical and professional skills in the workplace [1,2]. This provides students with the opportunity to actively build and incorporate theoretical and practice knowledge, to socialise into a professional practice community and to understand the complexities of health care delivery [3,4].

During clinical placements, students’ clinical learning is facilitated by clinical educators, also referred to as ‘preceptors’ or ‘clinical supervisors’ [5]. While various models of supervision are used within the allied health professions [6,7], clinical educators are generally responsible for facilitating the acquisition of profession-specific skills while students are out in the field [8]. During this time, the relationship between the clinical educator and student is considered fundamental to the success of the learning opportunity [3,9,10]. To this end, a number of studies have attempted to define various skills, attitudes and practices that pertain to an ideal clinical educator and in doing so, have defined many characteristics to which clinical educators might aspire [9,11-14]. This work generally implies that the presence of an educator with such characteristics is a major factor in ensuring a valuable clinical learning experience [9].

University programs also expect students to be actively engaged in contributing to their experience and determining appropriate learning outcomes. This is aligned with the principles and theory of adult learning. In the clinical context, this requires that educators collaborate with students’ to identify their learning needs, ensure they are provided with opportunities to be self-directed and develop a learning experience that supports the students’ level of interest. This enhances the opportunity for better student engagement and development of a relationship for learning. For the student, this requires that they are well prepared in terms of foundational knowledge and attitudes at the commencement of practice education. This infers that they accept a share of the responsibility for planning and preparing for the learning experience. Attention to preparation allows students’ to take full advantage of the clinical opportunity by making sense of their experiences. To achieve this, health professional curricula are generally sequenced to ensure that students are prepared for clinical learning prior to a specific clinical experience.

In the clinical setting, supervision of a poorly prepared student with an inadequate knowledge base adds to the demanding nature of the supervisory relationship. Thus, the onus for creating effective and successful relationships during clinical learning also falls to students who must present themselves as competent, professional and well prepared [15]. Being well prepared will also maximise the learning a student gains from the placement. So what characteristics distinguish a competent, professional and well-prepared student to a clinical educator? A small body of work from Cross in the UK in the late 1990s evaluated the perceptions of university staff and clinical educators on the desirable and undesirable attributes of undergraduate physiotherapy students on clinical placement [10,15,16]. Desirable attributes included good communication skills, eagerness to learn, empathy and having a good knowledge base while undesirable attributes included being unsafe, unwilling to learn, arrogant and unprofessional [10,15].

Since this work, professional practice and entry-level education has evolved with a greater focus on interprofessional collaboration as well as a focus on the acquisition of generic rather than profession-specific attributes [17-19]. In addition, this body of work evaluated physiotherapy students; no similar evaluation of student characteristics in other allied health professions has been published. Thus, the primary aim of this interprofessional project was to ascertain clinical educators’ views on the key characteristics that, in their opinion, demonstrate that an allied health student is well prepared at the start of a clinical placement. In this study, allied health students included students from occupational therapy, physiotherapy and speech pathology.

## Methods

An observational approach using the Delphi technique that involved sequential on-line questionnaires interspersed by controlled feedback was used to gain consensus of opinion among clinical educators experienced with supervising occupational therapy, physiotherapy and speech pathology students [20]. Controlled feedback was provided by presenting summaries of the data from each round to participants with the process continuing until group consensus is achieved [21]. The Delphi technique is a widely used and accepted method for achieving consensus of opinion on a topic area solicited from experts within a field [20,22,23].

There were a number of advantages that made the Delphi technique suitable for exploring the issue of student preparedness for clinical learning opportunities [22]. First, it had the advantage of maintaining anonymity among respondents while allowing time for participants to consider their response. The anonymity minimised the possibility that a dominant group member or group pressure for conformity may influence the outcome as may occur in a face-to-face meeting [21,23]. Second, the technique allowed involvement of participants from diverse geographical locations and clinical backgrounds through the use of on-line and email communication. This was important as the sample consisted of allied health professionals located throughout Queensland in metropolitan, regional and remote areas. Third, the ability to use statistical analysis techniques allowed for objective and impartial analysis and summarisation of collected data [23]. For these reasons, the Delphi method has been used commonly in educational research as a valuable method of exploring underlying assumptions leading to differing judgements [10]. Approval for this study was obtained from the Human Research Ethics Committees of The University of Queensland and Queensland Health.

Two to three rounds of data collection are generally sufficient to elicit rich feedback and to reach consensus [21]. Once consensus is reached, further rounds are not needed. It was envisaged that three rounds would be sufficient to reach consensus but if consensus was reached after two rounds, the process could be terminated. Participants were to be provided feedback in the form of group responses from each previous round.

### Formation of the expert panel

The research team agreed to focus on the inclusion of clinical educators who engaged with students during block placements or who would be working with the same group of students over a number of weeks and for whom the learning relationship was a factor. Clinical educators who facilitated observational/one off experiences were not included in the expert panel. Thus, the expert panel consisted of clinical educators involved in the provision of clinical education to students enrolled in entry-level occupational therapy, physiotherapy and speech pathology programs at The University of Queensland during the preceding two years (2009/10). All clinical educators listed on The University of Queensland’s clinical educator mailing list were invited to participate. The list included only allied health staff with a designated clinical education role directly responsible for the supervision and assessment of students’ professional skills. All educators working with The University of Queensland are provided with training and support in the facilitation of student learning through the provision of regular clinical education workshops. An initial email was sent requesting involvement in the study. If clinical educators did not wish to be involved, they were requested to respond to this effect and were subsequently removed from the email list.

### First round questionnaire

The first questionnaire was drafted and edited by all members of the project team. The questionnaire underwent several iterations during development and was finally piloted on three clinical educators considered to be representative of the sample population. Following further revisions, the first round questionnaire consisted of two sections. The first section of the questionnaire focused on item generation by asking one open-ended question. Here, participants were asked to describe what they believed were the key characteristics that indicate that a student is prepared for a clinical placement and ready to learn. The second section of the questionnaire sought demographic information such as gender, age, years experience and area of expertise. A link to the on-line questionnaire was emailed to participants along with relevant information about the study. Participants were informed that by accessing the link and completing the questionnaire they were providing their informed consent. Two reminder emails were sent to all prospective participants during a six-week period.

### Second round questionnaire

The open-ended comments received for the first round were subject to a thematic framework analysis by the project team that used a staged approach [24]. Each project member read and re-read the comments and then coded the interesting features of the data before collating into potential themes drawing on the study objectives. In a face to face meeting, the team consisting of members of each profession (occupational therapy, physiotherapy and speech pathology) discussed different themes and reach agreement on theme title. Each team member had evaluated responses from one of the three professions prior to the meeting. Then the project leader developed characteristics within each theme based on responses with similar meaning. This was then viewed by the whole team and amendments made where disagreement in interpretation occurred. Responses were discarded if they were incomprehensible or had no relationship to the study objectives.

The outcome was six themes:

1. Theme 1 Knowledge and Understanding - denoted responses relevant to students’ demonstrating knowledge and understanding of related theory, processes and tasks.

2. Theme 2 Willingness – denoted responses relevant to students willingness to engage, assist, learn and practice.

3. Theme 3 Professionalism – denoted responses relevant to students’ demonstration of professionals skills and behaviours

4. Theme 4 Communication and interaction – denoted responses relevant to students’ demonstration of communication and interactive ability.

5. Theme 5 Personal attributes- denoted responses relevant to students’ personal attributes such as their personality traits.

6. Theme 6 Skills – denoted responses that were relevant to the students demonstration of various professional and interpersonal skills.

Under each theme, there were a number of characteristics based on the open-ended responses. Each team member was responsible for categorising the characteristics under one or two themes. These were then agreed upon through email and verbal discussion. The final questionnaire asked participants their opinion of the relative value of these characteristics as representative of a student who, at the beginning of a placement, was prepared and ready to learn. A seven-point Likert Scale was used (1 = not important, 2 = slightly important, 3 = somewhat important, 4 = moderately important, 5 = important, 6 = very important, 7 = extremely important) as seven point scales have been found to be more reliable than five-point scales [25]. The questionnaire was then emailed to participants who had responded to the first round. Again, two reminder emails were sent to non-respondents.

### Analysis

The responses to the first round were subject to a thematic analysis and demographic data were entered into Microsoft Excel for descriptive analysis. Data from the second questionnaire were reported as the means (SD) of the seven point Likert Scale. In addition, the interquartile deviation (IQD) for responses was calculated. The interquartile range is the absolute value of the difference between the 75th and 25th percentiles, with smaller values indicating higher degrees of consensus. An IQD of ≤ 1.00 has been identified as an indicator of consensus [26]. However, Rayens and Hahn (2000) suggested using a secondary criterion such as the percentage of generally positive respondents to questions with an IQD ≤ 1.00 to indicate agreement [27]. Commonly, a cut off of 70% generally positive respondents (5–7 on the Likert scale) means that if a factor has an IQD ≤ 1.00 and ≥ 70% of the respondents provided a positive response to this factor then it can be considered that consensus has been achieved. Finally, Cronbach’s alpha, a coefficient of reliability was used as a measure of the level of consistency of opinion among the respondents for each theme in the second round of the questionnaire. For comparing scales, alpha values of greater than 0.8 are regarded as good while those greater than 9 are regarded as excellent [28].

## Results

The first questionnaire was emailed to a total of 636 clinical educators in occupational therapy, physiotherapy and speech pathology. A total of 258 (40.56%) responded after reminder emails. There were an equal number of clinical educators from occupational therapy, physiotherapy and speech pathology with most working in metropolitan centres and 57 per cent of the sample having more than 5 years experience in clinical education (Table 1). The second questionnaire was sent to only those participants who had responded to the first questionnaire. A total of 161 clinical educators completed the second round. This represents a 62.40 per cent retention rate from round one and an overall response rate of 25.31 per cent of the original population.

**Table 1:** Demographics of the participants responding to the first round

		**Occupational therapy (n = 85)**	**Physiotherapy (n = 88)**	**Speech Pathology (n= 85)**
**Gender**				
Females	N (%)	81 (95.29)	59 (67.05)	80 (94.12)
Years since commenced practice	Mean (SD)	14.13 (10.47)	15.60 (10.39)	11.46 (10.05)
**Place of work**				
Metropolitan (population > 100,000)	N (%)	68 (80.0)	70 (79.55)	56 (65.88)
Provincial (population 25,000 – 99,999)	N (%)	12 (14.12)	15 (17.05)	19 (22.35)
Regional (population < 25,000)	N (%)	4 (4.71)	4 (4.55)	8 (9.41)
Regional/Rural (population < 5,000)	N (%)	3 (3.53)	1 (1.36)	2 (2.35)
**Years involved in clinical education**				
1-4 years	N (%)	34 (40.0)	43 (48.86)	34 (40.0)
5-9 years	N (%)	25 (29.41)	26 (29.55)	21 (24.71)
10+	N (%)	26 (30.59)	19 (21.59)	30 (35.29)

The means and the IQD for each characteristic, along with the percentage of participants who agreed that the attributes were ‘important’, ‘very important’ or ‘extremely important’, are presented in Table 2. All factors reached consensus with IQD values equal to or less than 1. Of these, consensus was reached that five characteristics were not important (Table 2). Three were in the knowledge and understanding theme while two were in the professional attributes theme. For example, over 75 per cent of the sample perceived that it was not important that students demonstrated some understanding about the department or organisation where they will be undertaking the placement. Similarly, over 50 per cent perceived it as not important that a student demonstrates knowledge of other professions and their roles.

**Table 2:** Means, percentage ratings and the IQD for each characteristic (* characteristics considered not important)

**THEME: KNOWLEDGE AND UNDERSTANDING**	**Percentage >4**	**MEAN**	**IQD**
The student demonstrates sound theoretical knowledge in basic sciences	78	5.18	0.5
The student demonstrates a thorough knowledge of therapy practices relevant to the area*	52	4.42	0.5
The student knows how to access information when a gap in knowledge or need for further information is identified	91	5.97	0.5
The student demonstrates basic knowledge of the key features of common conditions	74	5.09	1
The student demonstrates some understanding about the department or organisation where they will be undertaking the placement*	25	3.66	0.5
The student demonstrates knowledge of basic treatment principles for common conditions*	68	5.06	1
The student demonstrates knowledge of forms of treatment that may be detrimental to a client	71	5.02	1
The student demonstrates knowledge of other professions and their roles*	48	4.22	1
The student has an understanding of own learning style	82	5.34	0.5
The student demonstrates knowledge of the clinical assessment tools their educator is using to assess them	75	5.14	0.5
**THEME: WILLINGNESS**			
The student is willing to work as a team with peers, colleagues and other health professionals	100	6.19	0.5
The student is willing to ask questions and clarify to ensure understanding	99	6.44	0.5
The student is willing to try new techniques	98	5.95	1
The student is willing to discuss and exchange ideas to maximise patient care	99	6.23	0.5
The student is willing to receive feedback/constructive criticisms	100	6.51	0.5
The student displays a willingness to take on board any appropriate requested task	97	6.14	0.5
The student is willing to stray from their comfort zone	84	5.41	0.5
The student is willing to adhere to positive workplace culture and routines e.g. tidying up, cleaning	96	5.93	1
The student is willing to take responsibility for their own learning	98	6.33	0.5
The student is willing to self evaluate	99	6.13	0.5
**THEME: PROFESSIONALISM**			
The student has a thorough understanding of the code of conduct and ethics for their profession	85	5.53	0.5
The student understands their role and is able to verbalise this	79	5.13	0.5
The student arrives at the placement on time	96	5.96	1
The student’s appearance is appropriate for the workplace and placement (e.g. hair, fingernails, jewellery)	96	5.89	1
The student is dressed appropriately for the placement (e.g. closed in shoes, uniform if appropriate, visible ID badge)	96	5.93	1
The student complies with professional matters such as confidentiality	99	6.70	0
The student attends each day having demonstrated appropriate follow up from previous day	94	5.86	0.5
The student makes appropriate contact with facility/educator prior to the placement commencing	88	5.70	0.75
The student is prepared for the first day having completed the appropriate pre-reading and bringing learning resources relevant for the clinical area(s)	84	5.57	0.75
The student displays ability to maintain professional boundaries with patients/clients	95	6.27	0.5
The student respectfully engages with people from a wide range of cultures and backgrounds	97	6.20	0.5
**THEME: COMMUNICATION AND INTERACTION**			
The student demonstrates effective communication and interpersonal skills (verbal, non-verbal and listening) with clients across the lifespan	89	5.75	1
The student is able to liaise with key stakeholders, such as organising appointments	73	5.03	1
The student is able to communicate professionally with members of the multidisciplinary team	87	5.53	0.5
The student demonstrates respectful and non-judgemental communication	97	6.19	0.5
The student has the capacity to adjust their interaction style to meet the needs of the audience, whether it be colleagues, clients or others	89	5.60	0.5
The student demonstrates effective written communication skills, in charts, letters and information for clients	81	5.26	0.5
**THEME: PERSONAL ATTRIBUTES**			
The student demonstrates enthusiasm and interest in the placement	92	5.79	0.5
The student shows initiative	97	5.86	0.5
The student is sensitive/empathetic to client's needs and concerns	99	6.01	1
The student has the ability to manage stress levels	87	5.36	0.5
The student demonstrates a desire to learn	99	6.26	0.5
The student demonstrates the ability to self reflect on performance, interactions and outcomes	93	5.95	1
The student has self awareness of own limitations and is honest about current level of knowledge & skills	97	6.04	1
The student demonstrates the ability to apply oneself	97	5.92	0.5
The student is attentive	97	5.90	0.5
The student is curious and asks questions	94	5.77	0.5
The student is proactive	94	5.78	0.5
The student is diligent	95	5.77	0.5
The student is self directed	91	5.69	0.5
The student is helpful	84	5.45	0.5
The student is polite	93	5.82	1
The student is creative*	63	4.73	1
The student is assertive *	60	4.65	1
**THEME: SKILLS**			
The student demonstrates time management skills e.g. use of a diary, to do lists	82	5.38	0.5
The student demonstrates organisational skills	85	5.45	0.5
The student has good verbal and written skills	90	5.57	0.5
The student demonstrates good observational skills	86	5.60	0.5
The student has research skills to find basic information to fill in existing knowledge gaps	90	5.69	0.5
The student has foundation skills for the area of practice	81	5.35	0.5
The student demonstrates social skills e.g. the ability to relate personably	93	5.81	1
The student demonstrates problem-solving skills	95	5.73	0.5

While there was agreement on 57 characteristics, educators tended to value characteristics within the themes of ‘willingness’, ‘professionalism’ and ‘personal attributes’ more than characteristics in the ‘knowledge and understanding’ theme (Table 2). Thus, views on student preparedness appear to be based on external professional traits, such as appropriate dress and appearance, and a willingness to be involved in learning and the placement rather than a specific level of knowledge and understanding.

Cronbach’s alpha for the second round of the Delphi process ranged between 0.85 and 0.93 (Table 3). This indicates a good to excellent degree of internal consistency in the responses for characteristics within each theme. As consensus was reached after the second round, there was no need for a third questionnaire.

**Table 3:** Cronbach’s Alpha for each theme

**Theme**	**Cronbach’s Alpha**
Knowledge	0.85
Willingness	0.89
Professionalism	0.86
Communication and interaction	0.90
Personal attributes	0.94
Skills	0.88

## Discussion

This consensus study is the first to identify a range of characteristics perceived by clinical educators as indicators of an allied health student who is prepared and ready for a clinical learning opportunity. Six themes with a total of 57 characteristics were identified as being important. By conducting this study, a set of attributes relating to behaviours has been compiled and could be provided to students to aid their preparation for clinical learning. The final list of characteristics represents the consensus opinion of 161 experts in clinical education in Queensland, Australia. Of these, just over half the sample had more than five years experience as a clinical educator. In addition, we used several analytical methods (inter-quartile deviation, percentage agreement scores) to determine when consensus was reached. Cronbach’s alpha, which ranged between 0.85-0.94 for the second round, should be considered substantial and is consistent with reliability scores obtained for validated scales in clinical use [29]. Our methodical and analytical approach, in addition to the solicitation of experienced clinical educators, ensures the developed list of attributes has content validity.

There are several interesting outcomes from this research. The first obvious finding is the large number of characteristics developed by the expert panel. However, some are not perceived to be as important as others. The three themes viewed as more important than others were ‘willingness’, ‘professionalism’ and ‘personal attributes’. For example, nine of 10 characteristics in the ‘willingness’ theme were viewed as important by over 90 per cent of participants. This contrasts to the theme ‘knowledge and understanding’ where only one of ten characteristics scored above 90 per cent. Thus, clinical educators’ views on preparedness appear based on external professional traits such as appropriate dress and appearance, along with a willingness to be involved in learning rather than a specific level of knowledge and understanding they portray commencing a placement. This may reflect the view that educators believe that knowledge takes longer to demonstrate or is developed in the context of clinical learning. Thus, it could be argued that perceptions of readiness to learn are represented by an initial phase that focuses on demonstrated external features of professional and interpersonal behaviours.

Second, the attributes developed by the expert panel were predominantly generic in nature. No attributes were raised that were profession-specific such as a particular clinical skill or assessment technique. This result is similar to that reported by Cross who identified eight constructs that were considered desirable for physiotherapy students on clinical placement: professional, abilities/persona, safety, communication, general disposition, knowledge base, approach to learning and commitment [15]. In our study, the focus on generic attributes appeared to occur irrespective of the discipline. This finding reaffirms the focus by universities on students’ development of generic attributes and also suggests that educators believe that profession specific skills are likely to be consolidated during clinical placement.

Third, supervisors in this study appear to be focused on students’ readiness to engage in the learning environment through their personal attributes, willingness and demonstration of knowledge. The results support the status of these students as adult learners to take responsibility for their learning and demonstrate their willingness to activity engage. To build on the advantage of situated learning, educators and universities need to promote clinical learning experiences with learners as part of the clinical environment rather than being temporary adjuncts [30]. The findings of this study promote understanding of how best to facilitate students’ transition into professional practice environments so they become more than a temporary adjunct.

A surprising outcome was the perception by our sample that it was not important for a student to demonstrate knowledge of other professions and their roles. Awareness of interprofessional practice is an important graduate skill [31]. Our findings suggest that clinical educators may have viewed work-based learning as the location where students learn about and from other professions. Alternatively, the judgements of clinical educators are influenced by their professional competency statements that feed into the assessment tools used to evaluate students during clinical education placements [31]. Across the professions, the assessment tools and overarching competency statements tend to encapsulate interprofessional engagement implicitly under an umbrella of descriptors such as professional communication and professional behaviour [32-34]. This explanation may account for the identification by clinical educators of traits such as ‘the student is willing to work as a team with peers, colleagues and other health professionals’ and ‘the student is able to communicate professionally with members of the multidisciplinary team.’

Another interesting finding is the absence of indicators relating to knowledge and use of technology. In a health and education environment where technology in the form of communication via e-health and telehealth systems is increasing in popularity, universities are addressing the use of technology for providing efficient and effective health outcomes [35]. A possible interpretation is that clinical educators believe that these skills are inherent in every student and that, therefore, they do not need to be stated. An alternative explanation is that educators do not consider knowledge of technology practices an important skill for clinical practice. The latter explanation disregards contemporary thought about health and education practice but further investigation and clarification with educators would shed light on this disparity.

This Delphi study had a few limitations. First, the sample of clinical educators was from one state in Australia and may not be generalisable to other states or countries. Second, while the response rates are comparable to other similar studies, our response rate of 40.6 per cent and 25.3 per cent in the first and second rounds may have led to self selection bias [10]. This means that the non-responders may have had systematically different responses than those who elected to respond. However, our retention rate between the two rounds suggests considerable interest in the topic by the responders. Third, there is an issue of whether or not the 'learned consensus' has validity beyond demonstrating the preconceptions of educators and encouraging students to 'play the game' by meeting these. Finally, while the educators were all involved in the provision of formal clinical block placements, there is the potential that each educator may have had different conceptions of teaching that may have impacted upon the results. To some degree, this was mitigated against by the Delphi methodology that encourages participants to reassess their initial judgments about the information provided in previous iterations. This approach created a list of characteristics that could be used by students to help them prepare for placements as it benchmarks the implicit views and consensus opinion of each of the three professions in Queensland. Further research is required to understand the basis for the views of the educators and whether there are differences in the perceptions between professions.

## Conclusions

The Delphi methodology allowed the development of a consensus-based list of attributes that clinical educators perceived identify a student who is well prepared for clinical placement. While the list of characteristics is long, the list should be useful to educators involved in the preparation of students for clinical placement. The findings should also be useful to curricula developers as this study suggests preparation of students to undertake clinical learning goes beyond the acquisition of knowledge, understanding and skills to, perhaps more importantly, include attention on the development of personal attributes and interpersonal skills. In addition, the information contained within the themes could provide a platform for discussions by professional bodies about the role of placement education, underpinning philosophies and pedagogies in addition to challenging assumptions about professionals. Further research to refine the items in the list and evaluate differences between the perceptions of each profession is needed.

## Competing interests

The authors report no declarations of interest.

## Authors’ contributions

All authors contributed to the concept and design, acquisition of data and interpretation of data. LC conducted the data analysis and wrote the first draft. All other authors were involved in revising the manuscript for important intellectual content and have given final approval of the version to be published.

## Pre-publication history

The pre-publication history for this paper can be accessed here:

http://www.biomedcentral.com/1472-6920/12/112/prepub
